# British and Foreign Medical Review

**Published:** 1845-02

**Authors:** 


We direct the attention of our readers to the British and Foreign Medical Review, which has been admirably conducted by the distinguished Editor, Dr. Forbes, for the last ten years. It is decidedly one of the ablest of the foreign periodicals, and contains an immense amount of valuable matter.

